# 

**Published:** 1904-11-26

**Authors:** 


### NOTICE TO CORRESPONDENTS.

All MSS., Letters, Books for Review, and other matters intended for the Editor should be addressed THE EDITOR, "THE HOSPITAL," THE HOSPITAL BUILDINGS, 28 & 29 SOUTHAMPTON STREET, STRAND, W.C.

The Editor cannot undertake to return rejected MS., even when accompanied by stamped directed envelope.

**Notice to Advertisers and Subscribers.**

All Advertisements, Orders for Copies of THE HOSPITAL, and Business Communications should be addressed to **THE MANAGER (Not to the Editor),** THE HOSPITAL, 28 & 29 Southampton Street, Strand, London, W.C.

The Cost of Subscription, post free, is as follows:—Per week, 3½d.; per quarter, 3s. 3d.; per half-year, 6s. 6d.; per annum, 13s. Foreign and Colonial:—Per annum, 19s. 6d., payable, in all cases, in advance.

**NOTICE.—Vol. XXXVI. of "The Hospital" (April to September 1904), in handsome cloth binding, is now on sale at the office of this Journal, price 10s. 6d. Cases for binding the Numbers contained in this Volume 2s. Index to Vol. XXXVI., 3½d., post free.**

**The Monthly part for December will be ready on Dec. 1. Price 1s., by post, 1s. 3d.**

### BOOKS RECEIVED.

J. Murray.

"Biochemistry of Muscle and Nerve." By W. D. Halliburton, M.D., F.R.S.

A. Constable and Co., Limited.

"The Surgery of the Diseases of the Appendix Vermiformis and the Complications." By Battle and Corner.

Swan Sonnenschein and Co.

"The Oxford and Cambridge Year Book, 1904." Oxford.

"The Oxford and Cambridge Year Book, 1904." Cambridge.

"Vegetarian and Simple Diet." By Colonel Kenney-Herbert.

"Education Through the Imagination." By J. McMillan.

J. Wright and Co., Bristol.

"An Introduction to Dermatology." By Norman Walker.

H. J. Glaisher.

"*X* Rays. Their Employment in Cancer and other Diseases. By Richard J. Cowen.

J. B. Lippincott Co.

"Orthodontia." By W. H. Jackson, M.D.

"International Clinics." Vol. III. Fourteenth Series.

### Medical Appointments.

**C. E. Abbott.** L.R.C.P.I., M.R.C.S.Eng., District Medical Officer of the St. Austell Union. **Donald Armour**, M.B., M.R.C.P.Lond., F.R.C.S.Eng., Surgeon to the Belgrave Hospital for Children. **C. T. U. Babst**, F.R.C.P&S., Certifying Factory Surgeon for the Wallsend District, Northumberland. **F. W. Bloomer**, M.R.C.S., L.S.A., Public Vaccinator to the Stapleford District of the Shardlow Union. **W. E. Brierley**, M.R.C.S., L.R.C.P., House Surgeon to the Women's and Children's Hospital, Leeds. **H. Davison**, L.R.C.P.I., L F.P.S.Glas., District Health Officer of the Spalding Union. **D. W. Inglis**, M.D.Glas., Certifying Factory Surgeon for the Jarrow District, Durham. **J. W. Killen**, M.B., B.S., R.U.I., First Assistant Medical Officer and Dispenser, Cleveland Street Asylum of the Central London Sick Asylum District. **W. Latham**, L.R.C.P.&S.Edin., L.F.P.S.Glas., Certifying Factory Surgeon for the London and North-Western Railway Company's Works at Earlestown in the Newton-le-Willows District, Lancashire. **L. E. Orton**, M.R.C.S., L.R.C.P., Certifying Factory Surgeon for the Bedworth District, Warwick. **J. M. Pooley**, M.R.C.S., L.R.C.P., First Assistant Medical Officer, Kensington Parish Infirmary. **R. Roper**, M.R.C.S., L.R.C.P., First Assistant Medical Officer at the St. Marylebone Infirmary. **Frank R. Seager**, L.R.C.P.&S.Edin., Civil Surgeon to Busreh, Turkish Arabia. **Winstan St. A. St. John**, M.R.C.S., L.R.C.P., Honorary Surgeon to the Derbyshire Hospital for Sick Children. **H. Stanley Turner**, M.R.C.S.Eng., L.R.C.P.Lond., Registrar to the Central London Ear and Throat Hospital. **Miss Veale**, M.B.Lond., Clinical Assistant to the Women's and Children's Hospital, Leeds. **L. Worts.** L.R.C.P.&S.Edin., District Medical Officer of the Leighton Buzzard Union.

**"The Hospital" Institutional Library.**

**"Hospitals and Asylums of the World." 4 Vols., with a Portfolio of Plans, complete £12 12s.**

**"Burdett's Hospitals and Charities, 1904." 5s. net.**

**"Burdett's Official Nursing Directory." 3s. net.**

**"Cottage Hospitals, General, Fever, and Convalescent." 10s. 6d.**

**"Uniform System of Accounts for Hospitals and Public Institutions." New Edition, 4s. net.**

**"Hospital Expenditure: The Commissariat." 2s. 6d.**

**"A Legal Handbook for the Use of Hospital Authorities." 2s. 6d.**

**"The Cottage Hospital Case Book or Register of Patients." 10s. and 12s. 6d.**

**All these are published by the Scientific Press, Ltd., and may be obtained through any bookseller, or direct from the publishers, 28 and 29 Southampton Street, Strand, London, W.C.**

### Medical and Administrative Appointments.

THE HOSPITAL FOR SICK CHILDREN, GREAT ORMOND STREET, LONDON, W.C.

Notice is hereby given that the office of RESIDENT MEDICAL SUPERINTENDENT will become vacant on December 18th, 1904.

Candidates for this post are invited to send in their applications addressed to the Secretary, with copies of not more than three testimonials given specially for the purpose, on or before 12 o'clock on WEDNESDAY, December 7th, 1904.

Candidates must be registered medical practitioners, and must have held a responsible hospital appointment.

The appointment is made for one year, but may be held, subject to annual re-election, for a period of three years.

Salary 100 guineas per annum, with Board and Residence in the hospital, and £5 washing allowance.

All candidates must be present and appear before the Joint Committees at their meeting on Thursday, December 8th, 1904, at 5 P.M. precisely.

Forms of application and copy of rules may be obtained from the Secretary.

By order of the Managing Committee,
JAMES McKAY, Acting Secretary.
November 8th, 1904. (2112)

ADDENBROOKE'S HOSPITAL, CAMBRIDGE.

SECRETARY-SUPERINTENDENT WANTED.

He will be required to live close to the Hospital, and to devote his whole time and energies to its service. He will be responsible for all secretarial business, for the keeping of the accounts, for the collection and increase of subscriptions, and for the order and general administration of the Hospital in all matters except the medical and surgical treatment of patients.

He will be required to find security for £500.

He will be paid a commencing salary of £250 per annum. Clerical assistance will be given.

It is desirable that he should commence his duties on the 26th December, 1904.

All applications, which should be accompanied by not more than three testimonials, must reach the Secretary not later than the 3rd December, 1904.

Canvassing will be treated as a disqualification.

Particulars may be obtained from the Secretary.

JOHN BONNETT, Secretary.
23 St. Andrew's Street, Cambridge.
November 16th, 1904. (2111)

### Books for Nurses.

THE SCIENTIFIC PRESS LTD.

### GENERAL NURSING TEXT-BOOKS.

**A HANDBOOK FOR NURSES.**
The best and latest Nursing Manual.
By J. K. WATSON, M.D.
Profusely Illustrated. Cloth gilt.
**5/-** net, **5/4** post free.

**NURSING: ITS THEORY AND PRACTICE.**
Being a complete Text-book of Medical, Surgical and Monthly Nursing.
By PERCY G. LEWIS, M.D. **3/6** post free.

**NURSING: ITS PRINCIPLES AND PRACTICE.**
By ISABEL ADAMS HAMPTON.
New and Enlarged Edition.
**7/6** net, **7/10** post free.

**PRACTICAL GUIDE TO SURGICAL BANDAGING AND DRESSINGS.**
By WM. JOHNSON SMITH, F.R.C.S.
Suitable size for the apron pocket.
Profusely Illustrated. Cloth. **2/-** post free.

**THE NURSES' PRONOUNCING DICTIONARY OF MEDICAL TERMS AND NURSING TREATMENT.**

New Edition. **2/-** post free.

**SURGICAL WARD-WORK AND NURSING.**
By ALEXANDER MILES, M.D.Edin.
Second Edition. With particulars and illustrations of the instruments used in various operations.
Cloth gilt. **3/6** net, **3/10** post free.

**FIRST AID TO THE INJURED AND MANAGEMENT OF THE SICK.**
An Ambulance Handbook and Elementary Manual of Nursing.
By E. J. LAWLESS, M.D., D.P.H. Illustrated.
**3/6** post free.

### SPECIAL NURSING TEXT-BOOKS.

*JUST OUT.*

**THE MODERN NURSING OF CONSUMPTION.**
By Dr. JANE WALKER.
Cloth boards. **1/-** net, **1/2** post free.

**OPHTHALMIC NURSING.**
By Dr. SYDNEY STEPHENSON.
New Revised Edition. Illustrated.
**3/6** net, **3/10** post free.

**NURSING IN DISEASES OF THE THROAT, NOSE, AND EAR.**
By Dr. P. MACLEOD YEARSLEY.
Illustrated. Cloth.
**2/6** post free.

**MENTAL NURSING.**
By Dr. WM. HARDING.
Bound in cloth. **1/-** post free.

**ART OF MASSAGE.**
By Mrs. CREIGHTON HALE.
Second Edition. Illustrated.
**6/-** post free.

**THE EXAMINATION OF THE URINE.**
By J. K. WATSON, M.D.
Cloth boards. Suitable size for the apron pocket.
**1/-** net, **1/1** post free.

**MASSAGE AND MEDICAL GYMNASTICS, INCLUDING THE SWEDISH AND SCHOTT MOVEMENTS.**
By Dr. GRAFSTROM.
Illustrated. Cloth. **2/6** post free.

**THE SCIENTIFIC PRESS, LTD.**

*Publishers of Nursing Text-Books, Case-Books, Charts, &c,*
**28 & 29 Southampton St., Strand, London, W.C.**
NEW CATALOGUE POST FREE ON APPLICATION.

